# Dietary pattern and nutrient intakes in association with non-communicable disease risk factors among Filipino adults: a cross-sectional study

**DOI:** 10.1186/s12937-020-00597-x

**Published:** 2020-08-03

**Authors:** Imelda Angeles-Agdeppa, Ye Sun, Keith V. Tanda

**Affiliations:** 1 Department of Science and Technology, Food and Nutrition Research Institute, Bicutan, Taguig, Philippines; 2 Nestlé Research Singapore Hub, Singapore, Singapore

**Keywords:** Dietary pattern, AHEI, NCD, Adult

## Abstract

**Background:**

This study evaluated the relationship between dietary quality and food patterns of Filipino adults and the rising prevalence of selected cardiometabolic non-communicable disease (NCD) risk factors.

**Methods:**

This is a cross-sectional study that examined the association of dietary pattern and NCDs using data collected in the 2013 National Nutrition Survey. A total of 19,914 adults aged 20 years and above were included in the analyses. The Alternative Healthy Eating Index (AHEI-2010) was used to characterize the dietary quality, and principal component analysis (PCA) was used to identify dietary patterns specific to the study population. Logistic regression models were applied to assess the association between the dietary pattern scores and selected cardiometabolic NCD indices including diabetes, hypertension, dyslipidemia and overweight and obesity with adjustment for potential confounders.

**Results:**

The mean AHEI-2010 score was 19.7 for women and 18.9 for men out of a total possible score of 100. Three major dietary patterns were identified through PCA: 1) meat and sweetened beverages (MSB); 2) rice and fish (RF) and 3) fruit, vegetables and snack (FVS). After adjustment for potential confounding factors, the AHEI pattern was associated with higher odds of overweight/obesity [extreme-tertile odds ratio (OR) 1.10, 95% confidence interval (CI) 1.02–1.21]. Subjects in the highest tertile of the MSB pattern had greater odds for overweight/obesity, diabetes, high total cholesterol, low HDL-cholesterol, high LDL-cholesterol, and high triglycerides (OR ranging 1.20 to 1.70, all *p*-value < 0.001). The RF pattern was associated with higher probability of overweight/obesity (OR 1.20, 95% CI 1.08–1.32) high LDL-cholesterol (OR 1.20, 95% CI 1.07–1.37), and less likelihood of diabetes (OR 0.87, 95% CI 0.77–0.98). The FVS pattern was associated with lower probability of overweight/obesity, diabetes, high triglycerides, and hypertension (OR ranging 0.85 to 0.90, all *p*-value < 0.05).

**Conclusions:**

Diet quality of Filipino adults is extremely poor. MSB and RF patterns were associated with a higher risk of cardiometabolic NCD indices, while FVS pattern was associated to lower risks. Identifying healthy and detrimental dietary patterns in the local diet could be informative for future local-based dietary recommendation and area-specific intervention programs.

## Background

Cardiometabolic syndrome (CMS) is a combination of metabolic dysfunctions mainly characterized by insulin resistance, impaired glucose tolerance, dyslipidemia, hypertension, and central adiposity. People with CMS are two times more likely to die from coronary heart disease and three times more likely to have a heart attack or stroke than those who do not have the syndrome. It is now known that central adiposity is a major contributor to increased cardiometabolic risk [1]. There are many challenges to bringing CMS risk factors under control. However, cardiometabolic programs and therapeutic strategies exist that combine diet and exercise prescriptions and focus on behavioral change to maximize success in reducing cardiometabolic risk factors. These programs have specific recommendations for calorie intake, nutrition, and ongoing cognitive and psychological assessments of habits and unhealthy behaviors [2].

In the Philippines, NCD have overtaken communicable diseases as the top cause of mortality wherein it is estimated to account for 67% of all deaths in 2016 [3]. The five major NCD in the Philippines in proportion to mortality are cardiovascular diseases (35%), cancers (10%), chronic respiratory diseases (6%), diabetes (4%), and other NCD (12%) [4]. Specifically, diseases of the heart and of the vascular system are the leading cause of mortality in the Philippines [5]. The National Nutrition Survey (NNS) conducted by the Food and Nutrition Research Institute (FNRI) in 2013 showed a large number of Filipinos at risk of selected cardiometabolic NCD factors. Risk factors assessed in the NNS include hypertension, obesity, high cholesterol, and diabetes [6]. In 2014, there were 16 for every 1000 Filipino patients admitted due to a medical condition wherein hypertension was possibly the most common etiology factor [7]. Moreover, in the past decade it has been observed that there is a steady increase in the prevalence of high fasting blood glucose (FBG) from 3.4% in 2003 to 5.6% in 2013, and the prevalence is even higher among Filipino adults residing in urban areas (6.4%).

Food, diet and nutritional status are important determinants of NCD. Poor dietary quality, in particular high salt intake, high saturated and *trans*-fatty acid intake, and low fruit and vegetable consumption coupled with sedentary lifestyle and stressful environment are some risk factors of CMS development [8]. The role of diet in the etiology of most NCDs is extremely important and considered a modifiable risk factor for NCDs [9]. The Philippines is at a high risk for a rise in NCDs as measured by selected CMS especially among adults since the pattern of consumption among this population group is associated with the consumption of processed food laden with sugar, salt and fat, drinking alcohol, snacking between meals, eating while distracted and sedentary lifestyle [10]. In addition, it has been recognized that dietary patterns rather than single nutrients are stronger predictors of NCD risks, and should be the focus for NCD prevention.

Limited data exist in the Philippines with regards to the local dietary patterns and their associations with NCD. Thus, this study evaluated the relationship between dietary quality and food patterns of Filipino adults and the rising prevalence of selected cardiometabolic NCD risk factors. Through the use of the Alternative Healthy Eating Index (AHEI-2010), which is based on foods and nutrients predictive of chronic disease risk, we could assess the quality of typical Filipino diet. A data-driven approach was also employed to understand major dietary patterns in the population. Using data collected in NNS 2013, dietary patterns derived from both approaches were studied in association with major NCD biomarkers, with the aim to identify potential protective or detrimental dietary patterns using local data that could guide future dietary intervention strategies appropriate and applicable in the Philippines.

## Materials and methods

### Study design and populations

This study used the data from the 2013 NNS. This is a cross-sectional, population-based survey that characterizes the health and nutritional status, foods consumption and dietary patterns of the Filipino population. The survey used a multi-staged stratified sampling design to represent all 80 provinces of the country covering both urban and rural areas. The first stage of sampling was the selection of Primary Sampling Unit (PSU). A PSU is a barangay or contiguous barangay with at least 500 households. It then follows the selection of Enumeration Areas (EA), a contiguous area in a barangay with 150–200 households. The final sampling unit is the household. The survey protocol was approved by the Ethics Committee of FNRI, and all study participants provided written informed consent.

### Data collection

#### Demographic and socio-economic data

Demographic and socio-economic information were collected from the 2013 NNS survey participants, including age, gender, area of residence, marital status, and education. Wealth status of participants was defined by proxy indicators including household possession of vehicles, appliances, materials used for housing construction and sanitation facilities. Scores obtained from principal component analysis were used to define wealth quintiles as poorest, poor, middle, rich and richest.

#### Dietary data

The 2 non-consecutive 24-h (24 h) dietary recall was conducted by registered nutritionist-dietitians through face-to-face interviews in households using structured questionnaires. The interviewer recorded all foods and beverages consumed on the previous day from the moment when they woke up until they went to sleep in the evening. The amount of foods and beverages consumed was estimated using household measures (cups, tablespoons and pieces) or through weighing of food samples. The weights of foods were converted to as purchased values using a portion-to-weight list for common foods compiled by FNRI. If the food was a dish, the interviewee was asked to describe the ingredients of the recipe or name the dish or recipe. The nutrient content of these composite foods were determined by breaking down the different ingredients in the recipe and each was calculated based on INFOODS Guidelines [11].

#### Derivation of dietary patterns

We adapted the AHEI-2010 with a priori defined scoring rules to assess the dietary quality of Filipino adults. The scoring criteria for AHEI-2010 were described in detail elsewhere [12]. Briefly, dietary quality was assessed by the intake per day of vegetables, fruit, whole grains, sugar sweetened beverages, nuts and legumes, red/processed meat, fish, alcohol, percentage of energy for polyunsaturated fatty acids (PUFA), and sodium. The intake of each dietary component was scored from 0 (poor diet) to 10 (optimal diet). In the original AHEI-2010 there is an inclusion of *trans*-fat in the scoring, but this was excluded in our study due to unavailability of *trans*-fat information in the Philippines Food Composition Table. Therefore, the AHEI-2010 score in our study was the sum of the scores from 10 foods and nutrients components and the total score ranged from 0 to 100 (Table 1).

**Table 1:** AHEI-2010 scoring criteria and mean component and total scores among adult men and women.^a^

Component	Criteria for minimum score(0)	Criteria for maximum score(10)	AHEI-2010 Women	AHEI-2010 Men
Vegetables, *servings/d*	0	≥5	0.66 ± 1.0	0.75 ± 1.2
Fruit, *servings/d*	0	≥4	0.56 ± 1.4	0.50 ± 1.4
Whole grains, *g/d*			0.20 ± 1.1	0.17 ± 1.1
Women	0	75		
Men	0	90		
Sugar-sweetened beverages, *servings/d*	≥1	0	2.9 ± 4.1	2.4 ± 3.9
Nuts and legumes, *servings/d*	0	≥1	1.9 ± 3.6	2.0 ± 3.8
Red/processed meat, *servings/d*	≥1	0	1.7 ± 2.9	1.3 ± 2.6
Fish, *serving/d*	0	≥0.2857	6.0 ± 4.5	6.3 ± 4.5
Alcohol, *drinks/d*			0.04 ± 0.6	0.11 ± 0.9
Women	≥2.5	0.5–1.5		
Men	≥3.5	0.5–2.0		
PUFA, *% of energy*	≤2	≥10	0.72 ± 0.8	0.46 ± 0.6
Sodium, *mg/d*	Highest decile	lowest decile	5.1 ± 3.1	4.9 ± 3.2
Total	0	100	19.7 ± 7.9	18.9 ± 8.1

^a^ Adapted AHEI-2010; Abbreviations: AHEI: alternative healthy eating index; PUFA: polyunsaturated fatty acids

Principal component analysis (PCA) was used to extract dietary patterns of Filipino adults. Thirty-five major non-overlapping food groups were included in the PCA after variable standardization. The resulting components (dietary patterns) represent combinations of foods consumed by the participants. The number of components retained was based on eigenvalues (> 1), the scree plot, and factor interpretability. Varimax rotation was applied in order to obtain the simplest factor structure with improved interpretability. The coefficients defining the linear combinations after the rotation are called factor loadings and represent the correlations of each food group variable with the dietary component. A factor score was produced for each individual participant for each of the dietary components identified. Prior to PCA, a Kaiser-Meyer-Olkin test of sampling adequacy (0.5015) and a Bartlett test of sphericity (*p* < 0.001) was performed to assess whether the factor model as a whole was significant. Table 2 are the three components or dietary patterns which were obtained: 1) meat and sweetened beverages pattern (MSB); 2) rice and fish pattern (RF) and 3) fruit, vegetables and snack pattern (FVS)*.*

**Table 2:** Principal loadings of three major dietary patterns identified among Filipino adults.^a^

Food Groups	Meat and Sweetened Beverages Pattern	Rice and Fish Pattern	Fruit, Vegetables, and Snack Pattern
Meat (mainly pork and poultry)	0.478		
Sweetened beverages	0.4721		
Rice, noodles & pasta (mainly rice)		0.5688	
Oils (mainly coconut oil)		0.4626	
Fish & shellfish		0.3935	
Eggs & egg dishes		0.2746	
Other grains (mainly corn grits)		−0.4187	
Fruit			0.3761
Nut/pea/bean-based mixed dishes (mainly fried green pea)			0.3412
Sugar, syrups, preserves, jams, jellies (mainly sugar)			0.3188
Fat (mainly coconut cream)			0.3037
Savory snacks (e.g. corn chips, potato chips, fish cracker)			0.2824
Vegetables			0.2775
Non-alcoholic beverages (mainly coconut water, pure coffee, tea)			0.2682

^a^Extraction method: Principal component analysis (PCA) with varimax rotation. Only factor loadings > 0.25 or < −0.25 are shown in the table

#### Anthropometric data and non-communicable diseases biomarkers

Weight and height of respondents were measured using an electronic calibrated portable stadiometer (SECA) (SECA 217, Hamburg, Germany) and digital double window weighing scale (SECA 874, Hamburg, Germany). Both weight and height measurements were collected twice but a third measurement was taken when two measurements were greater than 0.5 kg or cm. The mean of the 2 measurements were recorded correspondingly. Body mass index (BMI) was computed as weight (in kg) divided by the square of height (in meter). Chronic energy deficiency (CED), overweight (OW) and obesity (OB) were determined using World Health Organization (WHO) definition: BMI < 18.5 for CED; Normal: 18.5–24.99; OW: 25.0–29.99 and OB: > = 30 kg/m^2^ [13].

Systolic and diastolic blood pressure (BP) measurements were collected by trained nurses prior to blood extraction using a non-mercurial sphygmomanometer (A&D Um-101TM) and stethoscope in compliance with the Department of Health (DOH) Administrative Order No. 2008–0021. For every measurement, the mean of two readings taken at least two minutes apart was recorded. Blood samples were collected by trained registered medical technologists (RMT) from the study participants after 10–12 h overnight fasting. Blood samples were first collected using vacutainer tubes with Lithium Heparin for fasting blood glucose and plain tubes for lipids profile, after which they were stored on ice and later centrifuged to separate plasma, alter packed, labelled and frozen at − 20 °C until ready for analysis in the laboratory. Fasting blood glucose and blood lipids profile (total cholesterol, LDL-cholesterol, HDL-cholesterol and triglycerides) were analyzed using enzymatic colorimetric method with Roche COBAS Integra and Hitachi 912.

Clinical cut-offs were used for each of the biomarkers in the study. Hypertension was defined as systolic BP > =140 mmHg and/or diastolic BP > =90 mmHg according to the 8th Joint National Committee for the Detection, Diagnosis, Treatment and Follow-up of Hypertension [14] Fasting blood glucose was classified based on [15]: < 110 mg/dL as normal, 110–125 mg/dL as impaired fasting glucose (IFG), and > =126 mg/dL as diabetes. Lipid profile which includes total cholesterol (< 200 mg/dL as desirable, 200–239 mg/dL as borderline high, and > =240 mg/dL as high), LDL-cholesterol (< 130 mg/dL as desirable, 130-159 mg/dL as borderline high, and > 160 mg/dL as high), HDL-cholesterol (< 40 mg/dL as low, 40–59 mg/dL as borderline, and > =60 mg/dL as desirable), and triglycerides (< 150 mg/dL as desirable, 150–199 mg/dL as borderline, and > =200 as high/very high) was assessed using the criteria from Adult Treatment Panel (ATP) III Classification [16].

### Statistical analyses

The three PCA-derived dietary pattern factor scores as well as the AHEI-2010 scores were categorized as tertiles according to their distribution in the studied population. Descriptive statistics including means, standard errors (SE) and percentages were used to summarize clinical, social demographics and lifestyle of the participants by tertiles of the dietary pattern scores. Logistic regression analyses were used to test for associations between tertiles of the four dietary pattern scores (independent variables) and the selected CMS (dependent variables). The multivariable model (model 2) was additionally adjusted for total energy intake, age, sex, smoking status, drinking status, urbanity, and wealth status. Trend test across the three tertiles was assessed by modeling the median of each tertile as a continuous variable. Missing data in each variables were excluded in the analysis. All data were analyzed using STATA (version 13; Stata Corp., College Station, TX, USA). The level of significance was set at *P < 0.05.*

## Results

For this study, a total of 19,914 adults aged 20 years and above were included in the analyses (men: *n* = 10,001 and women: *n* = 9913), with a mean of age of 45.7 yrs. old.

Mean AHEI-2010 score in the studied Philippines adults population was 19.7 for women and 18.9 for men out of a total possible score of 100 (Table 1). This suggested an overall poor quality of diet in the general population. A mean score of 28.2 even in the highest tertile of AHEI-2010 (Table 3) could barely be considered a healthy eating group of subjects. Such lack of variation in the data limited the potential of this hypothesis-based healthy dietary pattern score to differentiate various subgroups of the population. Correspondingly, most of the demographic characteristics of the study participants did not differ significantly across the three tertiles of AHEI-2010 (Table 3). On the other hand, greater differences were observed across the tertile distribution of the three PCA-derived dietary patterns (Table 3). Respondents consuming a MSB pattern (highest tertile) are more likely to be younger, urban residents, from the rich and richest wealth quintiles, non-smoker, and currently drinking alcohol. The highest tertile of RF pattern are more likely to be younger, males, urban residents, from the rich and richest wealth quintiles, currently smoking and drinking alcohol. Subjects in the highest tertile of the FVS patterns are more likely to be from the richest wealth quintile and less likely to be currently smoking or drinking.

**Table 3:** Demographic and health characteristics by tertile of the four dietary patterns^a^

	AHEI-2010			MSB			RF			FVS		
Subject characteristics	T1	T2	T3	T1	T2	T3	T1	T2	T3	T1	T2	T3
Age (y)	44.2 ± 0.20	46.5 ± 0.20	46.6 ± 0.19	48.7 ± 0.19	47.1 ± 0.20	41.5 ± 0.18	49.0 ± 0.21	45.8 ± 0.19	42.5 ± 0.18	44.5 ± 0.19	46.5 ± 0.19	46.4 ± 0.19
Gender (%)												
Male	53.2	50.4	47.1	53.7	44.7	52.2	36.6	47.5	66.6	52.9	48.7	49.1
Female	46.8	49.6	52.9	46.3	55.3	47.8	63.4	52.5	33.4	47.1	51.3	50.9
Residence (%)												
Rural	54.2	57.9	52.4	72	53.3	39.3	58.2	54.7	51.6	53.7	55.7	55.1
Urban	45.8	42.1	47.6	28	46.7	60.7	41.8	45.3	48.4	46.3	44.3	44.9
Wealth status (%)												
Poorest	19.2	21.3	21.3	19.8	21.9	20	18.6	21.5	21.6	21.3	21	19.4
Poor	20.8	22.1	20.5	26.7	21.7	15	22.4	21.2	19.8	22	22.4	19
Middle	20.9	21.8	17.8	33.7	18.1	8.7	24.6	18.7	17.2	21.1	19.7	19.8
Rich	18.6	18.5	20.9	12	21	25	17.3	19.4	21.4	18.7	19.5	19.8
Richest	20.5	16.4	19.5	7.8	17.3	31.3	17.1	19.2	20	16.9	17.4	22
Currently smoking (%)	28.7	27.6	24.7	30.1	24.1	26.7	23.7	24.7	32.6	29.1	26.7	25.1
Currently drinking (%)	50.2	48.6	48.5	46.7	45.4	55.2	40.9	47.1	59.3	53	46.5	47.8
BMI status (%)												
CED	12.3	12.8	10.1	14.5	11.8	8.9	15	11.8	8.6	11.8	12.2	11.3
normal	59.9	59.5	59.4	63.4	58.5	56.8	59.4	59.2	60.2	59	60.1	59.6
overweight	22.2	21.9	24.1	18.4	23.6	26.3	20.1	23	25	23.2	22.1	22.9
obesity	5.6	5.8	6.4	3.7	6.1	8	5.5	6	6.2	6	5.6	6.2
Hypertension (%)	24.4	25.5	25.8	25.8	27.3	22.6	26.7	25.5	23.5	25.4	25.6	24.8
Diabetes (%)	19.2	18.5	18.5	16.7	19.2	20.1	19.1	19	18	20.2	17.5	18.5
Total cholesterol > 240 mg/dL (%)	19	18.7	20.4	16.2	21.1	20.8	19.9	20	18.2	18.3	19.6	20.2
LDL-cholesterol > 160 mg/dL (%)	22.1	23.6	24.9	21.1	25.7	23.8	24.4	24.5	21.7	22.3	24	24.3
HDL-cholesterol < 40 mg/dL (%)	59.6	61.9	59.9	67.6	59.9	53.6	60.2	60.2	61	61.6	60.7	59.1
Triglycerides > 200 mg/dL (%)	21.5	20.6	20.7	18.5	21.2	23.2	18.4	20.6	23.7	21.7	20.3	20.7
AHEI-2010 Score	10.7 ± 0.04	19.0 ± 0.02	28.2 ± 0.1	20.6 ± 0.1	21.0 ± 0.1	16.4 ± 0.2	18.6 ± 0.1	19.7 ± 0.1	19.7 ± 0.1	18.9 ± 0.1	19.2 ± 0.1	19.8 ± 0.1

^a^Values are means ± standard errors or percentages. Abbreviations: AHEI: alternative healthy eating index; MSB: meat and sweetened beverages pattern; RF: rice and fish pattern; FVS: fruit, vegetables and snack pattern; BMI: body mass index; CED: chronic energy deficiency. ^b^Missing values (Blood Pressure:659, Total Cholesterol:2196, HDL-cholesterol:2196, LDL:2204, BMI:909)

The prevalence of abnormalities in selected cardiometabolic NCD risk factors did not differ significantly across the tertiles of AHEI-2010 score for most measures. In comparison, the highest tertile of MSB pattern was associated with lower prevalence of chronic energy deficiency, hypertension and low HDL-cholesterol, and higher prevalence of overweight, obesity, diabetes, high cholesterol, high LDL-cholesterol, and high triglycerides. The RF pattern was associated with lower prevalence of chronic energy deficiency, hypertension and high LDL-cholesterol, and higher prevalence of overweight, obesity, and high triglycerides. The FVS pattern was associated with lower prevalence of diabetes (Table 3).

The intake of energy, total fat and sodium in lowest tertile of AHEI pattern were higher than the intake in the highest tertile, while magnesium, potassium and vitamin C intakes were higher in the highest tertile than the intake in lowest tertile (Table 4). The highest tertile of MSB pattern was associated with higher intakes of energy, total fat, saturated fat (SFA), monounsaturated fat (MUFA), polyunsaturated fat (PUFA), protein, sugar, iron and sodium, and a lower average score of AHEI-2010. The intakes of energy, iron, calcium, magnesium, phosphorus, potassium, selenium, and niacin were higher in the highest tertile of the RF pattern than the lowest tertile. For the FVS pattern, the intakes of energy, calcium, fiber, folate, magnesium and potassium were higher than the intakes in the lowest tertile (Table 4).

**Table 4:** Nutrients intake of Filipino’s adults by tertile of the four dietary patterns

Nutrients	AHEI-2010			MSB			RF			FVS		
	T1	T2	T3	T1	T2	T3	T1	T2	T3	T1	T2	T3
**Macronutrients**												
Energy intake (kcal)	1763.1 ± 6.5	1652.9 ± 6.1	1682.3 ± 5.7	1608.7 ± 5.9	1578.2 ± 5.6	1911.5 ± 6.0	1365.2 ± 4.6	1659.5 ± 4.5	2073.6 ± 5.9	1639.8 ± 6.1	1642.7 ± 5.9	1815.8 ± 6.2
Total fat (g/d)	30.4 ± 0.2	25.7 ± 0.1	26.6 ± 0.1	20.6 ± 0.1	25.2 ± 0.1	36.9 ± 0.2	23.3 ± 0.1	26.8 ± 0.1	32.6 ± 0.2	26.4 ± 0.2	26.1 ± 0.1	30.2 ± 0.2
Saturated fat (g)	13.1 ± 0.1	11.6 ± 0.1	12.4 ± 0.1	9.6 ± 0.04	11.7 ± 0.1	15.8 ± 0.1	10.3 ± 0.1	12.0 ± 0.1	14.7 ± 0.1	12.0 ± 0.1	11.8 ± 0.1	13.2 ± 0.1
Monounsaturated fatty acids (g)	9.8 ± 0.1	8.5 ± 0.05	9.1 ± 0.04	7.0 ± 0.04	8.4 ± 0.04	11.9 ± 0.1	8.1 ± 0.05	9.0 ± 0.05	10.2 ± 0.1	8.8 ± 0.05	8.8 ± 0.04	9.8 ± 0.1
Polyunsaturated fatty acids (g)	4.6 ± 0.02	4.1 ± 0.02	4.2 ± 0.02	3.4 ± 0.02	4.0 ± 0.02	5.5 ± 0.02	3.9 ± 0.02	4.2 ± 0.02	4.8 ± 0.02	4.1 ± 0.02	4.1 ± 0.02	4.7 ± 0.02
Protein (g/d)	57.0 ± 0.2	54.8 ± 0.2	55.7 ± 0.2	52.4 ± 0.2	52.1 ± 0.2	63.0 ± 0.2	45.4 ± 0.2	54.6 ± 0.1	67.9 ± 0.2	55.7 ± 0.2	54.0 ± 0.2	57.8 ± 0.2
Carbohydrate (g/d)	307.3 ± 1.2	296.3 ± 1.2	301.1 ± 1.1	301.3 ± 1.2	283.7 ± 1.1	319.7 ± 1.1	240.2 ± 0.8	295.3 ± 0.8	369.1 ± 1.2	288.4 ± 1.1	294.1 ± 1.1	322.3 ± 1.2
Total sugars (g/d)	25.1 ± 0.1	23.2 ± 0.1	25.3 ± 0.1	23.1 ± 0.1	22.3 ± 0.1	28.2 ± 0.1	23.5 ± 0.1	24.5 ± 0.1	25.6 ± 0.1	19.5 ± 0.1	23.3 ± 0.1	30.8 ± 0.1
Dietary fibre (g/d)	8.4 ± 0.02	8.4 ± 0.03	8.9 ± 0.03	9.2 ± 0.04	8.0 ± 0.03	8.6 ± 0.03	8.1 ± 0.03	8.3 ± 0.03	9.3 ± 0.03	7.4 ± 0.02	8.4 ± 0.03	9.9 ± 0.04
**As percentage of total energy**												
Total Fat (%)	13.1 ± 0.01	13.3 ± 0.01	13.3 ± 0.01	13.1 ± 0.02	13.2 ± 0.01	13.3 ± 0.01	13.1 ± 0.01	13.2 ± 0.01	13.4 ± 0.01	13.5 ± 0.01	13.2 ± 0.01	13.0 ± 0.01
Protein (%)	71.4 ± 0.1	72.6 ± 0.1	72.3 ± 0.1	75.1 ± 0.1	72.5 ± 0.1	68.8 ± 0.1	72.5 ± 0.1	72.3 ± 0.1	71.6 ± 0.1	71.8 ± 0.1	72.6 ± 0.1	72.0 ± 0.1
Carbohydrates (%)	307.0 ± 1.1	299.6 ± 1.1	304.2 ± 1.0	308.8 ± 1.1	284.2 ± 1.0	317.8 ± 1.1	269.7 ± 1.0	297.0 ± 0.9	344.2 ± 1.1	281.3 ± 1.1	292.1 ± 1.0	337.5 ± 1.1
**Antioxidants**												
Vitamin C	20.9 ± 0.1	20.7 ± 0.1	22.1 ± 0.1	22.0 ± 0.1	20.0 ± 0.1	21.6 ± 0.1	21.3 ± 0.1	21.2 ± 0.1	21.1 ± 0.1	18.2 ± 0.1	20.8 ± 0.1	24.6 ± 0.1
Vitamin E	2.4 ± 0.01	2.2 ± 0.01	2.3 ± 0.01	2.1 ± 0.01	2.2 ± 0.01	2.6 ± 0.01	2.0 ± 0.01	2.2 ± 0.01	2.7 ± 0.01	2.1 ± 0.01	2.2 ± 0.01	2.5 ± 0.01
**B-vitamins**												
Thiamine (mg/d)	0.8 ± 0.01	0.7 ± 0.01	0.7 ± 0.01	0.7 ± 0.01	0.7 ± 0.01	0.8 ± 0.01	0.6 ± 0.01	0.7 ± 0.01	0.8 ± 0.01	0.7 ± 0.01	0.7 ± 0.01	0.8 ± 0.01
Riboflavin (mg/d)	0.7 ± 0.01	0.6 ± 0.01	0.7 ± 0.01	0.6 ± 0.01	0.6 ± 0.01	0.8 ± 0.01	0.6 ± 0.01	0.6 ± 0.01	0.7 ± 0.01	0.6 ± 0.01	0.6 ± 0.01	0.7 ± 0.01
Niacin (mg/d)	18.2 ± 0.1	18.1 ± 0.1	18.7 ± 0.1	17.4 ± 0.1	17.3 ± 0.1	20.2 ± 0.1	14.6 ± 0.1	18.1 ± 0.04	22.3 ± 0.1	18.4 ± 0.1	17.7 ± 0.1	18.8 ± 0.1
Vitamin B6 (mg)	1.5 ± 0.01	1.5 ± 0.01	1.5 ± 0.01	1.4 ± 0.01	1.4 ± 0.01	1.7 ± 0.01	1.2 ± 0.01	1.4 ± 0.01	1.8 ± 0.01	1.4 ± 0.01	1.4 ± 0.01	1.6 ± 0.01
Folate (DFE μg)	14.9 ± 0.1	13.5 ± 0.1	13.8 ± 0.1	11.3 ± 0.04	13.8 ± 0.05	17.1 ± 0.1	13.9 ± 0.1	14.0 ± 0.1	14.4 ± 0.1	13.9 ± 0.1	13.7 ± 0.1	14.6 ± 0.1
Vitamin B12 (mg)	3.5 ± 0.01	3.6 ± 0.01	3.7 ± 0.01	3.5 ± 0.01	3.5 ± 0.01	3.7 ± 0.01	3.2 ± 0.01	3.6 ± 0.01	4.0 ± 0.01	3.7 ± 0.01	3.5 ± 0.01	3.5 ± 0.01
**Bone-related nutrients**												
Calcium (mg/d)	832.5 ± 3.2	815.4 ± 3.0	834.6 ± 2.8	805.7 ± 3.1	775.9 ± 2.8	900.8 ± 3.0	648.7 ± 2.1	809.2 ± 2.0	1024.6 ± 2.8	813.5 ± 3.0	799.6 ± 2.9	869.3 ± 3.1
Phosphorus (mg/d)	169.7 ± 0.6	168.3 ± 0.6	175.2 ± 0.6	177 ± 0.6	159.4 ± 0.5	176.7 ± 0.5	147.2 ± 0.5	167.1 ± 0.5	198.9 ± 0.5	157.8 ± 0.5	166.5 ± 0.5	188.9 ± 0.6
Magnesium (mg)	8.4 ± 0.03	7.8 ± 0.03	8.0 ± 0.03	7.5 ± 0.03	7.5 ± 0.03	9.3 ± 0.03	6.9 ± 0.02	8.0 ± 0.02	9.4 ± 0.03	7.6 ± 0.03	7.8 ± 0.02	8.9 ± 0.03
Vitamin D (mg)	2.8 ± 0.01	2.9 ± 0.01	3.0 ± 0.01	2.8 ± 0.01	2.9 ± 0.01	3.1 ± 0.01	2.6 ± 0.01	2.9 ± 0.01	3.3 ± 0.01	3.0 ± 0.01	2.9 ± 0.01	2.9 ± 0.01
**Other micronutrients**												
Vitamin A (μg RE/d)	433.2 ± 1.5	424.6 ± 1.4	433.9 ± 1.4	430.3 ± 1.5	410.3 ± 1.4	451.1 ± 1.4	411.7 ± 1.5	424.4 ± 1.4	455.6 ± 1.3	404.4 ± 1.4	427.2 ± 1.4	460.1 ± 1.5
Iron (mg/d)	181.8 ± 0.9	172.4 ± 0.9	182.8 ± 0.9	180.8 ± 1.0	166.8 ± 0.8	189.4 ± 0.8	189.2 ± 1.1	167.1 ± 0.7	180.7 ± 0.7	149.8 ± 0.8	181.3 ± 0.9	205.9 ± 0.9
Zinc (mg)	5.9 ± 0.03	5.4 ± 0.02	5.5 ± 0.02	5.0 ± 0.02	5.2 ± 0.02	6.7 ± 0.02	4.7 ± 0.02	5.5 ± 0.02	6.6 ± 0.02	5.5 ± 0.02	5.4 ± 0.02	5.9 ± 0.02
Sodium (mg/d)	854.4 ± 2.9	706.1 ± 2.8	691.3 ± 2.4	656.3 ± 2.8	729.8 ± 2.5	865.6 ± 2.7	704.2 ± 2.8	741.7 ± 2.7	805.7 ± 2.9	723.9 ± 2.7	740.1 ± 2.8	787.6 ± 3.0
Potassium (mg)	1198.2 ± 3.8	1186.4 ± 3.7	1262.2 ± 3.7	1217.8 ± 3.9	1126.5 ± 3.4	1302.5 ± 3.6	1082.1 ± 3.5	1190.8 ± 3.2	1373.8 ± 3.6	1117.9 ± 3.3	1167.0 ± 3.3	1361.9 ± 3.9
Selenium (mg)	99.0 ± 0.4	93.1 ± 0.3	93.9 ± 0.3	87.9 ± 0.3	90.0 ± 0.3	108.2 ± 0.3	78.7 ± 0.3	93.8 ± 0.3	113.6 ± 0.3	95.1 ± 0.3	92.9 ± 0.3	98.0 ± 0.3

Values shown are mean ± SE of usual nutrient intakes. *MSB* Meat and sweetened beverages pattern, *RF* Rice and fish pattern, *FVS* Fruit, vegetables and snack pattern

Logistic regression analyses results of selected cardiometabolic NCD risk factors across tertiles of the 4 dietary patterns are provided in Table 5. After adjustment for various potential confounding factors, the AHEI pattern was associated with higher odds of overweight/obesity [odds ratio for extreme tertile comparison: 1.1, 95% CI: 1.02, 1.21]. The MSB pattern was associated with higher odds of overweight/obesity [1.3, 95% CI: 1.21, 1.47], diabetes [1.20, 95% CI: 1.10, 1.36], high total cholesterol [1.4, 95% CI: 1.29, 1.62], low HDL-cholesterol [1.7, 95% CI: 1.41, 2.10], high LDL-cholesterol [1.30, 95% CI: 1.15, 1.43], and high/very high triglycerides [1.30, 95% CI: 1.16, 1.43]. The RF pattern was associated with higher probability of overweight/obesity [1.20, 95% CI: 1.08, 1.32], high LDL-cholesterol [1.20, 95% CI:1.07, 1.37], and less likelihood of diabetes [0.87, 95% CI: 0.77, 0.98]. The FVS pattern was associated with lower probability of overweight/obesity [0.85, 95% CI: 0.77, 0.92], diabetes [0.88, 95% CI: 0.80, 0.97], high triglycerides [0.90, 95% CI: 0.81, 1.00], and hypertension [0.88, 95% CI: 0.81, 0.96].

**Table 5:** Multivariate adjusted odds ratio for non-communicable disease biomarkers by tertiles of four dietary patterns

	AHEI-2010				MSB				RF				FVS			
	T1	T2	T3	P-Trend	T1	T2	T3	P-Trend	T1	T2	T3	P-Trend	T1	T2	T3	P-Trend
**BMI (Overweight/Obese vs Normal)**																
Model 1	Ref	1.0 (0.92, 1.08)	1.1 (1.02, 1.19)	0.012	ref	1.5 (1.34, 1.87)	1.1 (1.05, 1.22)	< 0.001	ref	1.1 (1.05, 1.22)	1.2 (1.11, 1.30)	< 0.001	ref	0.93 (0.86, 1.01)	0.98 (0.91, 1.06)	0.948
Model 2	Ref	1.1 (0.97, 1.15)	1.1 (1.02, 1.21)	0.016	ref	1.3 (1.19, 1.42)	1.3 (1.21, 1.47)	< 0.001	ref	1.1 (1.03, 1.23)	1.2 (1.08, 1.32)	0.001	ref	0.89 (0.82, 0.97)	0.85 (0.77, 0.92)	< 0.001
**FBG (Diabetes vs Normal)**																
Model 1	Ref	0.96 (0.88, 1.05)	0.96 (0.88, 1.04)	0.368	ref	1.2 (1.08, 1.30)	1.3 (1.15, 1.37)	< 0.001	ref	0.99 (0.91, 1.08)	0.93 (0.85, 1.01)	0.091	ref	0.84 (0.77, 0.91)	0.88 (0.82, 0.98)	0.074
Model 2	Ref	0.97 (0.88, 1.07)	0.95 (0.86, 1.04)	0.282	ref	1.1 (1.03, 1.27)	1.2 (1.10, 1.36)	< 0.001	ref	0.99 (0.90, 1.10)	0.87 (0.77, 0.98)	0.016	ref	0.84 (0.76, 0.92)	0.88 (0.80, 0.97)	0.043
**Total cholesterol (High vs Desirable)**																
Model 1	Ref	0.99 (0.89, 1.09)	1.1 (1.04, 1.25)	0.006	ref	1.5 (1.36, 1.66)	1.5 (1.33, 1.63)	< 0.001	ref	1.0 (0.93, 1.12)	0.89 (0.82, 0.99)	0.021	ref	1.1 (1.0, 1.21)	1.1 (1.04, 1.26)	0.01
Model 2	Ref	0.96 (0.86, 1.07)	1.0 (0.93, 1.15)	0.474	ref	1.4 (1.23, 1.53)	1.4 (1.29, 1.62)	< 0.001	ref	1.1 (1.01, 1.25)	1.1 (0.99, 1.29)	0.073	ref	1.0 (0.94, 1.17)	1.0 (0.92, 1.13)	0.85
**HDL-cholesterol (Low vs Desirable)**																
Model 1	Ref	0.90 (0.76, 1.07)	1.1 (0.92, 1.28)	0.308	ref	1.5 (1.22, 1.77)	2.2 (1.86, 2.63)	< 0.001	ref	0.96 (0.81, 1.14)	0.90 (0.76, 1.07)	0.245	ref	1.0 (0.84, 1.20)	1.1 (0.96, 1.35)	0.11
Model 2	Ref	0.88 (0.74, 1.06)	1.1 (0.89, 1.27)	0.408	ref	1.2 (1.03, 1.51)	1.7 (1.41, 2.10)	< 0.001	ref	1.0 (0.85, 1.23)	1.0 (0.81, 1.25)	0.971	ref	0.93 (0.78, 1.11)	1.1 (0.90, 1.29)	0.273
**LDL-cholesterol (High vs Desirable)**																
Model 1	Ref	1.1 (1.01, 1.21)	1.2 (1.09, 1.30)	< 0.001	ref	1.4 (1.23, 1.48)	1.2 (1.10, 1.32)	0.002	ref	1.0 (0.92, 1.09)	0.83 (0.75, 0.90)	< 0.001	ref	1.1 (1.03, 1.23)	1.2 (1.05, 1.26)	0.006
Model 2	Ref	1.0 (0.95, 1.16)	1.1 (0.97, 1.18)	0.181	ref	1.3 (1.15, 1.41)	1.3 (1.15, 1.43)	< 0.001	ref	1.2 (1.05, 1.29)	1.2 (1.07, 1.37)	0.002	ref	1.1 (0.95, 1.16)	1.0 (0.93, 1.14)	0.662
**Triglycerides (High/Very High vs Desirable)**																
Model 1	Ref	0.93 (0.84, 1.01)	0.95 (0.86, 1.04)	0.297	ref	1.2 (1.11, 1.34)	1.4 (1.28, 1.54)	< 0.001	ref	1.2 (1.06, 1.28)	1.4 (1.31, 1.57)	< 0.001	ref	0.91 (0.83, 0.99)	0.94 (0.86, 1.02)	0.328
Model 2	Ref	0.97 (0.88, 1.07)	0.98 (0.88, 1.07)	0.626	ref	1.3 (1.4, 1.39)	1.3 (1.16, 1.43)	< 0.001	ref	1.0 (0.90, 1.10)	0.96 (0.85, 1.08)	0.454	ref	0.96 (0.87, 1.06)	0.90 (0.81, 1.0)	0.044
**Blood pressure (Hypertension vs Normal)**																
Model 1	Ref	1.1 (0.98, 1.14)	1.1 (0.99, 1.16)	0.805	ref	1.1 (0.99, 1.17)	0.84 (0.77, 0.91)	< 0.001	ref	0.94 (0.86, 1.02)	0.84 (0.78, 0.91)	< 0.001	ref	1.0 (0.93, 1.09)	0.96 (0.89, 1.05)	0.356
Model 2	Ref	0.99 (0.91, 1.09)	1.0 (0.91, 1.09)	0.919	ref	1.2 (1.10, 1.31)	1.1 (1.01, 1.22)	0.082	ref	1.0 (0.95, 1.13)	1.02 (0.92, 1.14)	0.708	ref	0.93 (0.86, 1.02)	0.88 (0.81, 0.96)	0.008

*AHEI* alternative healthy eating index, *MSB* Meat and sweetened beverages pattern, *RF* Rice and fish pattern, *FVS* Fruit, vegetables and snack pattern, *T1* tertile 1, *T2* tertile 2, *T3* tertile 3, *ref*. reference group, *BMI* body mass index, *FBG* fasting blood glucose. Values are odds ratios (95% confidence intervals). Model 1 was unadjusted logistic regression model. Model 2 was adjusted for total energy intake, age, sex, smoking status, drinking status, urbanity, and wealth status. ^b^Missing values (Blood Pressure: 659, Total Cholesterol: 2196, HDL-cholesterol:2196, LDL:2204, BMI:909)

## Discussion

This study evaluated the relationship between dietary quality and food patterns of Filipino adults and the rising prevalence of selected cardiometabolic NCD risk factors. Dietary quality was derived from the national food consumption survey adopting the AHEI-2010 pattern as standard. The respondents in this study reported poor overall diet quality as illustrated by the very low mean score of AHEI-2010 of less than 20 out of 100. This is very low compared with the findings in many other countries: Brazilian population had a mean adapted HEI-2015 of 45.7; among Americans, the mean AHEI-2010 was 52.4 for men and 47.6 for women out of 110; the Chinese had a mean AHEI-2010 of 42.2 for men and 43.8 for women out of 80; and the finding among Singaporeans revealed that the median quintile range of AHEI-2010 was 48.1–51.6 out of 110 total score [12, 17–19]. Very low consumption of vegetables, fruits, and whole grains were the main contributing factors for the poor quality of diet, and these could be due to several reasons: high price, poor availability, low accessibility and possible contamination of pesticides, lack of knowledge on the benefits of these foods, and no time to cook especially among working adults [20, 21]. In a previous study, better diet quality is seen in women compared with men due to higher awareness and better nutrition knowledge of women than men and several studies also point out that women seek nutrition counselling more frequently than men do [22]. In this present study only a slight difference in AHEI is seen among women (19.7) and men (18.9). This insignificant difference can be attributed to the varied modes of acquiring information about nutritious diet on different social media platforms.

Due to lack of variability in the studied sample using the hypothesis-based approach, AHEI-2010 score was not associated with many socio-demographic characteristics and the selected CMS. Therefore, we explored dietary patterns which could be potentially more meaningful to the local diet with a data-driven approach, PCA. Three major dietary patterns were identified, a meat and sweetened beverages pattern (MSB), a rice and fish pattern (RF), and a fruits, vegetables and snack pattern (FVS).

Our respondents who consume a MSB and RF patterns (highest tertile) are more likely to be younger, urban residents, and from the rich and richest wealth quintile. This is in conformance with an earlier study which revealed that dietary patterns differ between urban and rural areas due to differences in educational attainment, financial resources, and access to healthier foods [23, 24]. Furthermore, urban areas have higher accessibility to a wide range of processed and traditional high-sugar, high-fat snack foods and beverages [25]. The Food and Agriculture Organization statistics also showed that fish consumption in urban areas stood at 14.5 kg per capita per year compared to 11 kg per capita per year in rural areas, this is in line with our finding that the RF pattern are more likely to be consumed by urban residents. Also in our study, respondents who are in the highest tertile of the FVS patterns are more likely to be from the richest wealth quintile. This is in agreement with the study in Korea where fruit consumption is associated with higher income and educational level [26]. The same findings were seen in Australiaand China [27, 28].

In terms of association with cardiometabolic NCD risk factors, the MSB pattern were associated with a higher risk of various metabolic disorders including overweight and obesity, diabetes, and dyslipidemia, possibly through higher intakes of energy, fat, sugar and sodium. The RF diet also showed an association with cardiometabolic risks. It has been found that fish and rice are contaminated with methylmercury (MeHg) when produced in polluted areas. The chemical form of MeHg in fish tissue has recently been identified as attached to the thiol group of the cysteine residues in fish protein [29], which are not removed and destroyed by any cooking or cleaning processes. Similarly rice cultivated in Hg contaminated areas can contain relatively high levels of MeHg [30–34] and the main route of human MeHg exposure is related to frequent rice consumption [32]. A body of evidence was developed that addresses potential associations between MeHg and a range of cardiovascular effects. These include cardiovascular disease (coronary heart disease, acute myocardial infarction (AMI), ischemic heart disease), blood pressure and hypertension effects, and alterations in heart rate variability [35, 36]. There are strong evidences for causal associations with cardiovascular disease, particularly AMI in adult men [37–40]. On the contrary, the FVS pattern was associated with lower risk of overweight, obesity, diabetes, dyslipidemia, and hypertension, which could be mediated through higher intakes of various beneficial nutrients including fiber, folate, calcium, potassium and magnesium.

A high consumption of sugar-sweetened beverages is evident in this study. Increased consumption of free sugars is particularly indicated in the form of sugar-sweetened beverages. Sugar-sweetened beverages usually contain added sugar such as sucrose or high fructose corn syrup. Every 330 ml or 12 oz. portion of sugar-sweetened carbonated soft drinks typically contains 35 g (around nine teaspoon) of sugars and provide approximately 140 kcal of energy, but generally with little value of other nutrients [41]. As part of an unhealthy dietary pattern, this may have an effect on increased blood sugar, LDL-cholesterol and triglycerides. Thus, poor diet contributes to the occurrence of a cluster of disorders known as the metabolic syndrome: abdominal obesity, hypertension, dyslipidemia, and disturbed metabolism of glucose or insulin [42]. The presence of the metabolic syndrome increases the risk of developing NCDs such as cardiovascular diseases, diabetes, chronic respiratory diseases, and cancer [43, 44].

The prevalence of cardiometabolic NCD risk factors continues to rise in the Philippines and this is compounded by the practice of unhealthy lifestyle behaviours. In 2013, the prevalence of high fasting blood glucose among adults was 5.6%, and this has increased to 7.9% in 2018 [45, 46].

Additionally, the prevalence remained high for elevated blood pressure (19.2%) (NNS 2018 data), total cholesterol (18.6%), LDL-cholesterol (21.9%) and triglycerides (17.7%). (NNS 2013 data) The key dietary components that lower cholesterol and triglycerides include increased consumption of fruits, vegetables, and whole grains instead of highly refined ones and plant-based protein [47, 48]. However, these are consumed in very small amounts in the studied population. Fruit and vegetable consumption of Filipino adults was only at 41 g and 114 g per capita respectively; further, only about 9.9% of the population were consuming whole grains.. In our study, the respondents who consumed a FVS pattern was observed to have an overall lower metabolic risk profile, which further corroborates the importance of promoting higher consumption of fruits, vegetables, and healthy snacks among the Filipino adults. Besides unhealthy diet, the prevalence of current smokers during the study period was 25.4%; binge drinkers was56.2%; and physical inactivity was 45.5%, and these numbers remained high in the latest national survey conducted in 2018. Promoting healthy lifestyle is indeed very much needed.

To our knowledge, our study is the first one to use recent nationally representative data to characterize the dietary patterns of adults in the Philippines. The utilization of both a priori defined index (AHEI-2010) and posteriori derived dietary patterns (PCA) provided complementary and comprehensive assessment of the Filipino dietary quality and food consumption patterns. However, this study has several limitations. Firstly, the dietary data collection using 24-h recalls is subject to measurement errors from the subjects’ recall and estimation of consumption portions. Secondly, the lack of *trans*-fat information in our food composition database limits our ability to assess *trans*-fat as a component of AHEI-2010 in association with cardiometabolic risk factors. Lastly, the cross-sectional design of the survey prohibits us from drawing conclusions about the causal relationship between the observed dietary pattern and the cardiometabolic NCD risk factors. Future prospective studies are warranted to corroborate the findings of the present study.

## Conclusions

This study first characterized the diet of Philippines adults using the AHEI-2010 method, which suggested overall poor quality of diet. Three major dietary patterns in the studied population were then identified using a data-driven approach (PCA). Diet quality of Filipino adults is extremely poor. Meat and sweetened beverages and rice and fish patterns were associated with a higher risk of all the cardiometabolic NCD indices, while a fruits, vegetables and snack pattern was associated to a lower risks of cardiometabolic risks. Identifying healthy and detrimental dietary patterns in the local diet could be informative for future local-based dietary recommendation and area-specific intervention programs.

## Data Availability

All data generated or analysed during this study are included in this published article and its supplementary information files.

## Abbreviations

AHEI: Alternative Healthy Eating Index
PCA: Principal Component Analysis
MSB: Meat and Sweet Beverages Pattern
RF: Rice and Fish Pattern
FVS: Fruit Vegetables and Snack Pattern
CMS: Cardiometabolic syndrome
IFG: Impaired Fasting Glucose
RMT: Registered Medical Technologists
NCD: Non-Communicable Diseases
NNS: National Nutrition Survey
FNRI: Food and Nutrition Research Institute
PSU: Primary Sampling Unit
EA: Enumeration Areas
FCT: Food Composition Table
OW: Overweight
OB: Obesity
BP: Blood Pressure
DOH: Department of Health
WHO: World Health Organization
ATP: Adult Treatment Panel
CED: Chronic energy deficiency
FBG: Fasting Blood Glucose
BMI: Body Mass Index
T1: Tertile 1
T2: Tertile 2
T3: Tertile 3
Ref: Reference group
RE: Retinol Equivalent
NE: Niacin Equivalent
α-TE: α-tocopherol equivalent
DFE: Dietary Folate Equivalent
MUFA: Monounsaturated Fatty Acids
PUFA: Polyunsaturated Fatty Acids
HDL: High-Density Lipoprotein
LDL: Low-Density Lipoprotein
SE: Standard Error
